# A Megavoltage CT Image Enhancement Method for Image-Guided and Adaptive Helical TomoTherapy

**DOI:** 10.3389/fonc.2019.00362

**Published:** 2019-05-10

**Authors:** Yaru Liu, Chenxi Yue, Jian Zhu, Haining Yu, Yang Cheng, Yong Yin, Baosheng Li, Jiwen Dong

**Affiliations:** ^1^Network-Based Intelligent Computing, University of Jinan, Jinan, China; ^2^Department of Radiation Oncology, Shandong Cancer Hospital, Shandong University, Jinan, China; ^3^Shandong Key Laboratory of Digital Medicine and Computer Assisted Surgery, The Affiliated Hospital of Qingdao University, Qingdao, China

**Keywords:** megavoltage CT, image guided radiotherapy, adaptive radiotherapy, tomotherapy, image enhancement, block matching, discriminative feature representation, irradiation dosimetry

## Abstract

**Purpose:** To propose a novel method to improve the mega-voltage CT (MVCT) image quality for helical TomoTherapy while maintaining the stability on dose calculation.

**Materials and Methods:** The Block-Matching 3D-transform (BM3D) and Discriminative Feature Representation (DFR) methods were combined into a novel BM3D + DFR method for their respective advantages. A phantom (Catphan504) and three serials of clinical (head & neck, chest, and pelvis) MVCT images from 30 patients were acquired using the helical TomoTherapy system. The contrast-to-noise ratio (CNR) and edge detection algorithm (canny) was employed for image quality comparisons between the original and BM3D + DFR enhanced MVCT. A simulated rectangular field of 6 MV X-ray beams were vertically delivered on the original and post-processed MVCT serials of the same CT density phantom, and the dose curves on both serials were compared to test the effects of image enhancement on dose calculation accuracy.

**Results:** In total, 466 transversal MVCT slices were acquired and processed by both BM3D and the proposed BM3D + DFR methods. Compared to the original MVCT image, the BM3D + DFR method presented a remarkable improvement in terms of the soft tissue contrast and noise reduction. For the phantom image, the CNR of the region of interest (ROI) was improved from 1.70 to 4.03. The average CNR of ROIs for 10 patients from each anatomical group, were increased significantly from 1.45 ± 1.51 to 2.09 ± 1.68 for the head & neck (*p* < 0.001), from 0.92 ± 0.78 to 1.36 ± 0.85 for the chest (*p* < 0.001), and from 1.12 ± 1.22 to 1.76 ± 1.31 for the pelvis (*p* < 0.001), respectively. The canny edge detection operator showed that BM3D + DFR provided clearer organ boundaries with less chaos. The root-mean-square of the dosimetry difference on the iso-center passed horizontal dose profile curves and vertical percentage depth dose curves were only 0.09% and 0.06%, respectively.

**Conclusions:** The proposed BM3D + DFR method is feasible to improve the soft tissue contrast for the original MVCT images with coincidence in dose calculation and without compromising resolution. After integration in clinical workflow, the post-processed MVCT may be better applied on image-guided and adaptive helical TomoTherapy.

## Introduction

Helical TomoTherapy has two modes of operation: a treatment mode with 6 MV X-ray beams and a megavoltage CT (MVCT) imaging mode with 3.5 MV beams (1). MVCT image contains the body and bony structures which is suitable for setup verification. In the meantime, because of the relatively lower absorption dose than kilo-voltage cone-beam CT (CBCT) (2, 3), daily MVCT acquisition is valuable in providing clinical variation information to predict cancer prognosis and organs at risk (OAR) complications before each fractionated dose delivery.

Different from the kilo-voltage X-rays diagnostic CT, the MVCT includes more Compton effects, which is relatively independent to Z. Larger numbers of X rays pass through the human body and scatter on the detector (4), resulting in amplified doping noise and lowered soft tissue contrast. Therefore, the low image quality of MVCT not only increases the difficulty of depicting the variations of tumors and OAR, but also makes it hard to provide an accurate image registration for image guided radiotherapy (IGRT) and accurate delineation for adaptive radiotherapy (ART) (5). In order to improve the quality of MVCT images without degrading the contrast resolution, Lu et al. proposed an anisotropic diffusion filter algorithm (6). However, Lu's algorithm has limited smoothness at sharp edges while it has excessive smoothness at the borders, and it did not increase the soft tissue contrast of the MVCT. On the contrary, this anisotropic diffusion filter often reduces the contrast for the small features (7). A tensor framework was used to improve MVCT image quality (8), which is a novel reconstruction technique for full or undersampled projections. This technique is effective in reducing image noise as well as streaking artifacts due to view aliasing in reconstructed images, while image resolution loss is noticeable when noise variation is high in MVCT. A denoising and texture enhancement (DeTECT) method was proposed (7), which has more effects on MVCT noise reduction and soft tissue enhancement. However, DeTECT for MVCT enhancement presented the following issues: first, image denoising by nonlocal means may cause hallucinations or objects that did not exist before; second, the selection of imaging processing parameters in the DeTECT algorithm is generally empirical. An efficient way to optimize these parameters for a particular image sets is not currently available.

In this study, the discriminative feature representation (DFR) method was employed, which has been proven to be an effective post-reconstructed solution to low-dose CT (LDCT) enhancement (9). However, considering the clinical practice, MVCT may scan only a few transversal slices (shot longitudinal distance) to spare the patient additional absorption doses. Therefore, the traditional DFR method should be modified for the MVCT enhancement in order to process the images with less slice numbers than the thickness of the discriminative dictionary. The Block-Matching 3D-transform (BM3D) method was enrolled in this study to provide an image space extension (9, 11).

This study aims to propose a novel and flexible BM3D and DFR combined (BM3D + DFR) method to improve MVCT image qualities based on post-reconstructed transversal 2D images, without losing the density accuracy.

## Materials and Methods

MVCT images of one phantom (Catphan504) and 30 patients were acquired using the helical TomoTherapy system, with the same imaging protocols “acquisition pitch 8 mm/rotation, reconstruction interval 2 mm.” An MVCT image of patients were scanned around a tumor on the anatomical sites of the head & neck, chest and pelvis. Ten patients were enrolled in each of the three anatomical groups. The imaging matrix size was 512 × 512. Imaging reconstruction was performed using the TomoTherapy operation site with a standard filtered back projection algorithm.

### BM3D Algorithm

The BM3D algorithm was proposed by Dabov in 2006 (10). The concept of BM (block-matching) was to find the blocks that match the current reference block with the shortest Euclidean distance. It processed blocks within the 2D image in a sliding manner. The matched blocks were stacked together to form a 3D array (group). Due to the similarity between the blocks, the noise was effectively attenuated by the 3D contraction transform coefficients. Inverse 3D transformations generated block estimates. After repeating this process, the final image estimate was calculated as a weighted average of all overlapping estimates (10–12). This algorithm was enrolled in this study to extend each 2D slice from the MVCT serial into a 3D image space, to meet the requirements of the traditional DFR method.

### DFR Algorithm

The DFR algorithm was proposed by Chen in 2017 (9) for LDCT enhancement, which has also proven to be a concise and effective approach for other reconstructed CT image quality improvements. DFR assumed LDCT images as the superposition of desirable high dose CT (HDCT) 3D features and undesirable noise-artifact 3D features. Regarding the application of MVCT enhancement, high-quality kilo-voltage CT (KVCT, generally the simulated planning CT) and low-quality MVCT clinical images correspondingly took the place of HDCT and LDCT of a phantom image serial, to construct a discriminative feature dictionary in this study.

### BM3D and DFR Combination Algorithm

**Figure 1:**
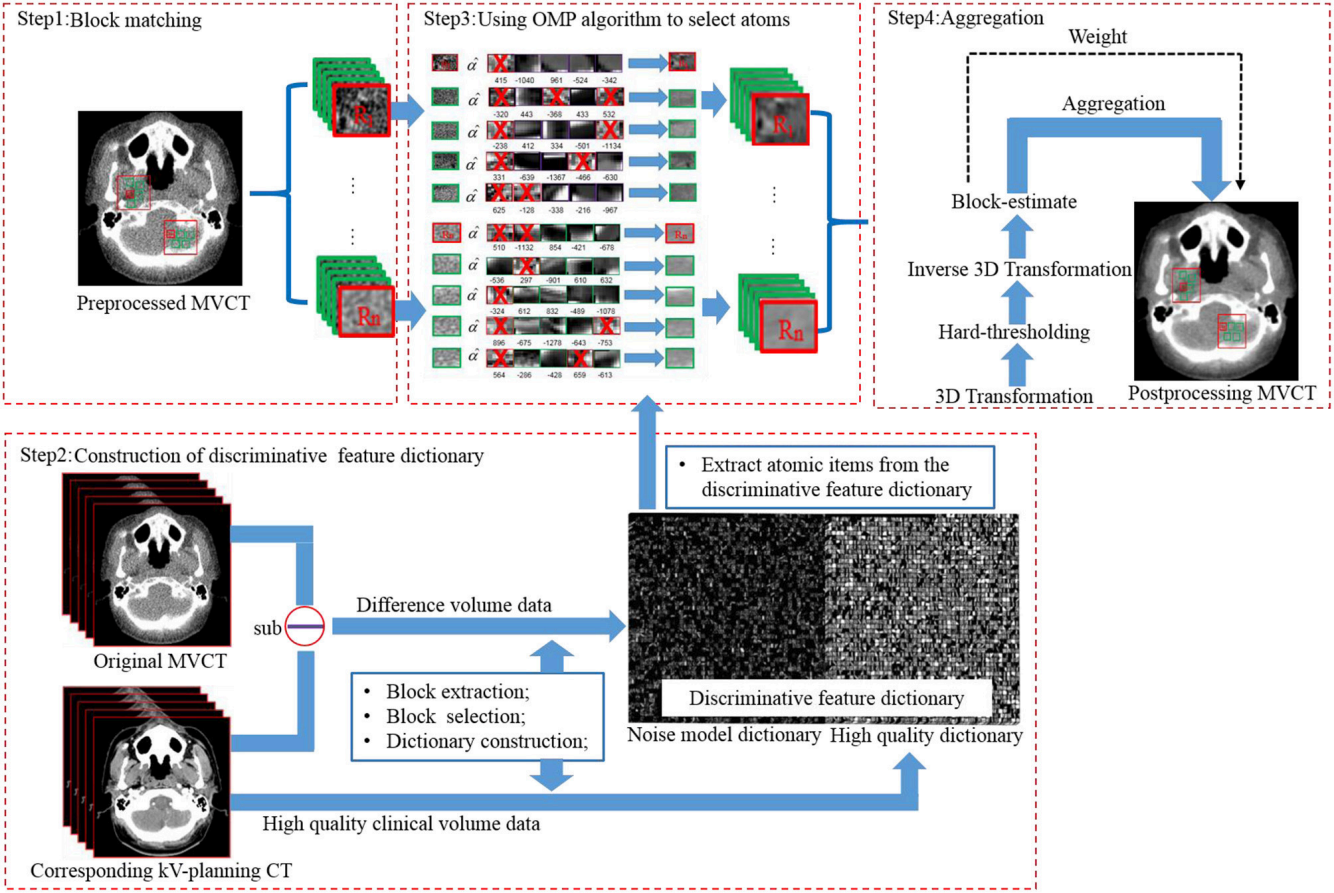
Flow chart of BM3D + DFR algorithm on MVCT enhancement.

The implementation flow of the BM3D + DFR method is shown in Figure 1 and explained as follows:

First, block matching (Figure 1 step1). Using a sliding manner in the L^*^L searching window, an MVCT image was divided into blocks with the size of $\sqrt{N_{block}}\times \sqrt{N_{block}}$. In the original MVCT image, a similarity search was performed for the blocks with higher similarity (shortest Euclidean distance) according to the hard-thresholding. The number of similar blocks was *N*_*number*_. To reduce the computational complexity of block-matching, the sliding interval was defined as Δ*d*. This process should be repeated to find multiple 3D arrays. A coarse pre-filtering method was employed to measure the similarity distance. The pre-filtering was realized by applying a normalized 2D linear transform on both the current reference block and the matched block. The obtained coefficients above were processed by hard-thresholding to achieve a similarity measurement according to Equation (1):

(1) $$\begin{array}{l} d\left({Z_{x}}_{R},Z_{x}\right)=\frac{\left\|\gamma^{\prime }\left(\left(\tau_{2D}^{ht}\left({Z_{x}}_{R}\right)\right)-\gamma^{\prime }\left(\tau_{2D}^{ht}\left(Z_{x}\right)\right)\right)\right\|}{{\left(N_{block}\right)}^{2}} \end{array}$$

where γ′is the hard-thresholding operator with threshold λ_2*D*_α, and ${\;\tau }_{2D}^{ht}\;$denotes the normalized 2D linear transform. Using the Equation (1), the result of block matching was a set that contains the coordinates of the blocks that are similar to*Z*_*x*_*R*__:

(2) $$\begin{array}{l} S_{x_{R}=\left\{x\in X|d\left({Z_{x}}_{R},Z_{x}\right)\leq \tau_{match}\right\}} \end{array}$$

(3) $$\begin{array}{l} b_{xyz}=A_{xyz}\left({S_{x}}_{R}\right) \end{array}$$

*d* is a similarity measurement between the reference patch *Z*_*x*_*R*__ and the matching patch *Z*_*x*_; τ_*match*_ is a similar maximum distance between two blocks. *S*_*x*_*R*__contains a set of the coordinates of the blocks that are similar to *Z*_*x*_*R*__. The variable symbol *A*_*xyz*_ is performed to extract the 3D blocks, and $A_{xyz}^{T}$ is a transpose matrix of *A*_*xyz*_.

Second is the construction of the discriminative feature dictionary (Figure 1 step 2). The discriminative feature dictionary *D* is composed by two discriminative sub-dictionaries, including the noise model dictionary (acquired from the difference between KVCT and MVCT clinical images) and the high-quality dictionary (acquired from high-quality KVCT clinical images). Feature blocks were extracted using a diagonal matrix and selected according to the “maximum features with minimum redundance” principle. More details can be found in the study of Chen et al. (9).

Third, is the use of the orthogonal matching pursuit (OMP) algorithm, to select atoms (Figure 1 step3). The stacked blocks (*N*_*block*_ × *N*_*block*_ × *N*_*number*_), with the same size as the atoms (*n*_*x*_ × *n*_*y*_ × *n*_*z*_) in the dictionary, were transformed into vectors. With DFR, each cell on the vector was replaced by the weighted items from the discriminative feature dictionary. Image blocks to be processed were sparsely represented by the atoms in dictionary using the OMP algorithm (13–15).

(4) $$\begin{array}{l} \alpha_{xyz}{=}_{\alpha_{xyz}}^{argmin}{\;\|b_{xyz}-D\alpha_{xyz}\|}_{2}^{2}{=}_{\alpha_{xyz}}^{argmin}{\;\|\left(A_{xyz}\left({S_{x}}_{R}\right)-dc_{xyz}\right)-D\alpha_{xyz}\|}_{2}^{2}\;\\ s.t.\|\alpha_{xyz}\|{}_{0}=\|\alpha_{xyz}^{hq},\alpha_{xyz}^{na}\|{}_{0}<C_{DFR},\forall \;x,y,z \end{array}$$

*D* represents the discriminative feature dictionary, and *C*_*DFR*_ denotes the sparsity constraint in Equation (4). Each sparse coefficient α_*xyz*_ is performed under the constraint. *b*_*xyz*_ represents a 3D array. *dc*_*xyz*_ represents the mean value of the block. Vector (*x, y*) denotes the axial plane, while *z* is the longitudinal axis, and thus *x, y, z* represents the three dimensions in an image volume space in Equation (4).

Fourth, aggregation (Figure 1 step4). A 3D transform was employed to further remove image noise from the post-processed image blocks.

(5) $$\begin{array}{l} {\;V}_{hq}=\frac{\sum_{xyz}\left({\hat{b}}_{xyz}^{hq}+dc_{xyz}\right)}{\sum_{xyz}\left(A_{xyz}^{T}A_{xyz}\right)}=\frac{\sum_{xyz}\left(D_{hq}\alpha_{xyz}^{hq}+dc_{xyz}\right)}{\sum_{xyz}\left(A_{xyz}^{T}A_{xyz}\right)} \end{array}$$

(6) $$\begin{array}{l} {\;Y}_{{S_{x}}_{R}}=\tau_{3D}^{{ht}^{-1}}\left(\gamma \left(\tau_{3D}^{ht}\left(V_{hq}\right)\right)\right) \end{array}$$

The volume *V*_*hq*_ is the denoised group; ${\hat{b}}_{xyz}^{hq}$ is the 3D block after dictionary representation. γ is a hard-thresholding operator with threshold λ_3*D*_α; ${\;\tau }_{3D}^{ht}$ denotes the normalized 3D linear transform, the 3D transform $\tau_{3D}^{ht}\;$of the 3D group consists of two transforms: a 2D transform denoted by $\tau_{2D}^{ht}$ (Bior 1.5) is applied on each block, and a 1D (Hadamard) transform, denoted by${\;\tau }_{1D}^{ht}$, is applied along the third dimension. *Y*__*S*_*xR*___ is the denoised group after 3D transformation and 3D inverse transformation.

In the sliding window, the selected reference blocks in one group were also referred as matching blocks in the other groups, which may be performed many times during block matching. The denoised image *Y*^*final*^ is computed as a weighted average of all those given by Equation (7):

(7) $$\begin{array}{l} Y^{\text{final}}=\frac{\sum_{x_{R}\epsilon X}\sum_{x_{m}\epsilon S_{x_{R}}}{\omega_{x_{R}}Y}_{x_{m}}^{x_{R}}}{\sum_{x_{R}\epsilon X}\sum_{x_{m}\epsilon S_{x_{R}}}\omega_{x_{R}}\lambda_{x_{m}}} \end{array}$$

where ω_*x*_*R*__ denotes the weights, λ_*x*_*m*__normalizes all weights to 1, and $Y_{x_{m}}^{x_{R}}\;$represents the block-wise estimation.

Parameter settings for the BM3D+DFR combination algorithm were set as follows:

Dictionary atom size *n*_*x*_ × *n*_*y*_ × *n*_*z*_: 8 × 8 × 5;
Sparsity constraint *C*_*DFR*_: 10;
Window width: 400 HU;
Window level: 40 HU;
Patch size *N*_*block*_ × *N*_*block*_: 8 × 8 pixels;
Number of similar blocks *N*_*number*_: 5;
Search window size *L* × *L* : 39;
Hard-thresholding τ_*match*_: 400;
Intervals Δ*d* : 3 pixels;

### Image Quality Evaluation

MVCT enhancement effects were compared between the original and the post-processed MVCT, including the MVCT enhanced by BM3D and the proposed BM3D + DFR in this study.

The visual enhancement effect is the primary criteria for radiation oncology physicians during IGRT registration and ART delineation on MVCT. Therefore, the original and post-processed MVCT images, including one phantom image serial of CatPhan504 and two clinical image serials of the head & neck and pelvis, were compared, respectively. Furthermore, considering that online ART requires quick delineation on MVCT slices when patients are lying on the treatment couch, the accurate automatic contouring on MVCT is necessary. Therefore, the edge detection algorithm “canny” was applied to the phantom and clinical MVCT serials to present the enhancement effects.

For the quantitative analysis, a contrast-to-noise ratio (CNR) (16, 17) was used to evaluate the improvement on the soft-tissue contrast. CNR is a measurement used for the change of image noise and contrast, where a larger value represents a higher image quality.

(8) $$\begin{array}{l} CNR=\frac{\sqrt{2}|A_{t}-A_{b}|}{\sqrt[{\frac{1}{2}}]{\left(\alpha_{t}^{2}+\alpha_{b}^{2}\right)}} \end{array}$$

where *A*_*t*_ and *A*_*b*_ are the mean pixel values of the target and the background region of interest (ROI), respectively. α_*t*_ and α_*b*_ are the standard deviations of the target and background ROI, respectively. No matter which anatomical site, the target and background ROI was always selected in the soft tissue regions in this study in order to assess the improvements on soft tissue contrasts.

Simulated irradiation beams on the Varian Eclipse (Varian Medical Systems, Palo Alto, CA) treatment planning system was used to test the stability of the CT number and the effects of the image enhancement on the dose calculation accuracy on post-processed MVCT. A rectangular field of 6MV X-ray beams were vertically delivered on both original and post-processed MVCT serials of the same CT density phantom. After dose calculation, the horizontal profile curve and vertical percent depth dose (PDD) curve on both serials were compared by plot comparison curves and the root-mean-square (RMS) of the dosimetry difference.

### Statistical Analysis

For the 30 patients of the three specific anatomical sites, CNR was calculated from every transversal slice of all the original and BM3D + DFR processed MVCT serials. The “average value ± standard deviation” of CNR was calculated on each anatomical site. Paired-sample *T*-tests (with 95% confidence interval) were used to compare the significance on the CNR between the original and post-processed MVCT. The statistical calculations were performed using the SPSS program, version 16.0.2 (SPSS Inc., Chicago, IL, USA).

## Results

For one phantom and all the 30 patients of the three anatomical sites, 466 transversal MVCT slices were acquired, including one center slice from the phantom, 323 slices from the head & neck, 51 slices from the chest and 91 slices from the pelvis. All these MVCT images were processed using both BM3D and the proposed BM3D + DFR methods.

### Visual Comparison of MVCT Image Enhancement Effect

**Figure 2:**
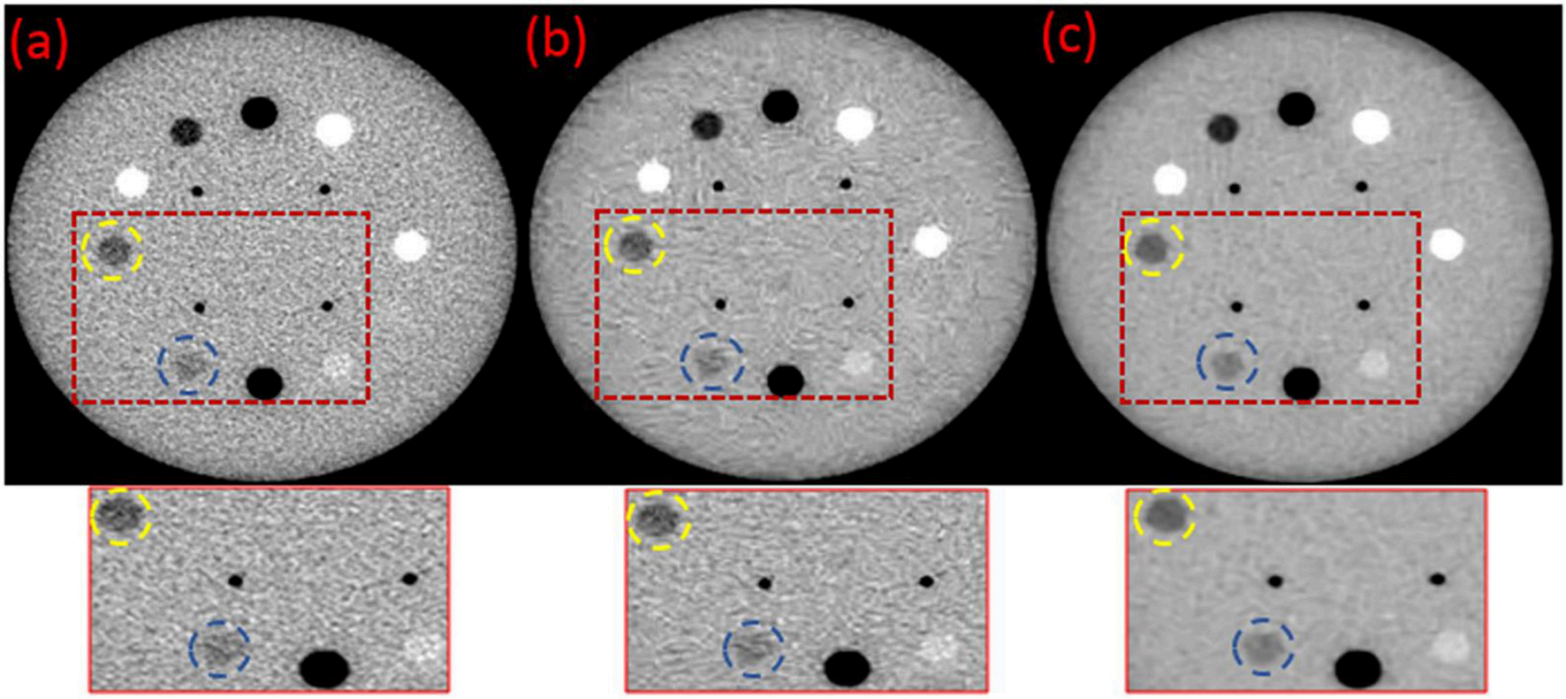
Original **(a)** and the post-processed MVCT images of Catphan504 by BM3D **(b)** and proposed BM3D+DFR method **(c)**.

Figure 2 shows a significant noise reduction and an improved resolution in image Figures 2b,c compared to image (Figure 2a) of the original MVCT. For image (Figure 2b), the noise was reduced by BM3D with some blurred structures. Using the proposed BM3D + DFR method, the noise in image (Figure 2c) was reduced, the boundaries of plugs were preserved and the contrast of plugs with similar densities were obviously improved. The yellow dotted circles represent the target regions and the blue dotted circles represent the background regions, which is utilized for CNR calculation.

**Figure 3:**
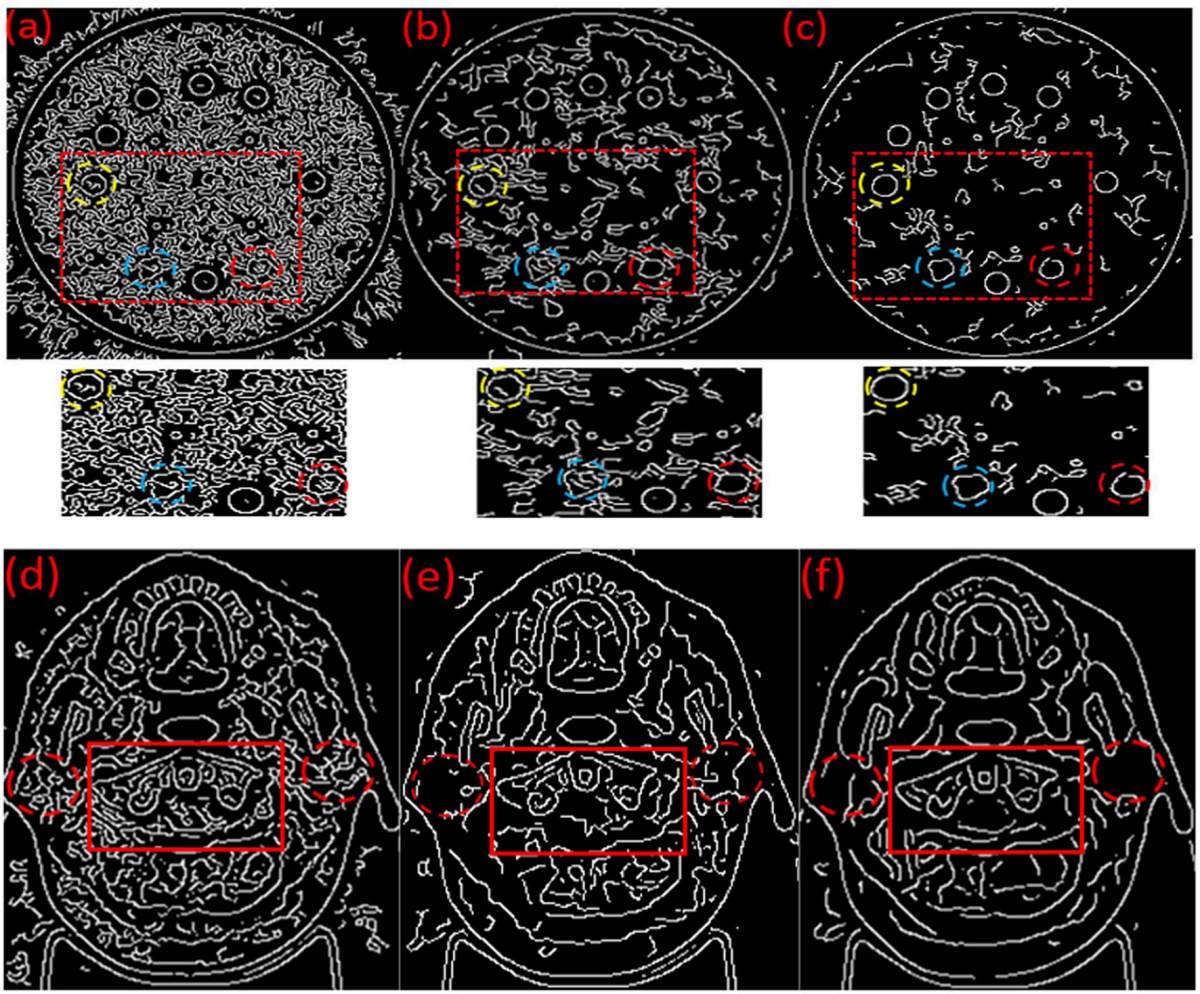
Automatic edges detection results on the original **(a,d)**, BM3D processed **(b,e)** and BM3D + DFR processed **(c,f)** MVCT serials of phantom and head & neck images.

The automatic edge detection results on the original MVCT images (Figures 3a,d), post-processed MVCT images using the BM3D (Figures 3b,e) and BM3D + DFR (Figures 3c,f) algorithms are shown in Figure 3. The edges of the plugs and organs in the circle selected in the original MVCT image are covered by a considerable amount of noise in Figures 3a,d. The edge detected on the post-processed MVCT images by BM3D and BM3D + DFR are encircled by decreasing noise. Especially when focusing on the three boundaries of the plugs marked by yellow, blue and red dotted circles on the phantom, the BM3D + DFR algorithm shows the best detection results with sharp edges and the least noise. Considering this, the edges of the organs on the clinical images are covered by plenty of noise in Figure 3d. While processed by BM3D + DFR, the edges of the bi-lateral parotid glands and cervical vertebra in Figure 3f are more conspicuous than the original as well as those processed by BM3D. The BM3D + DFR even allows for the edge of the spinal cord to be detected leaving very little noise in the parotids.

**Figure 4:**
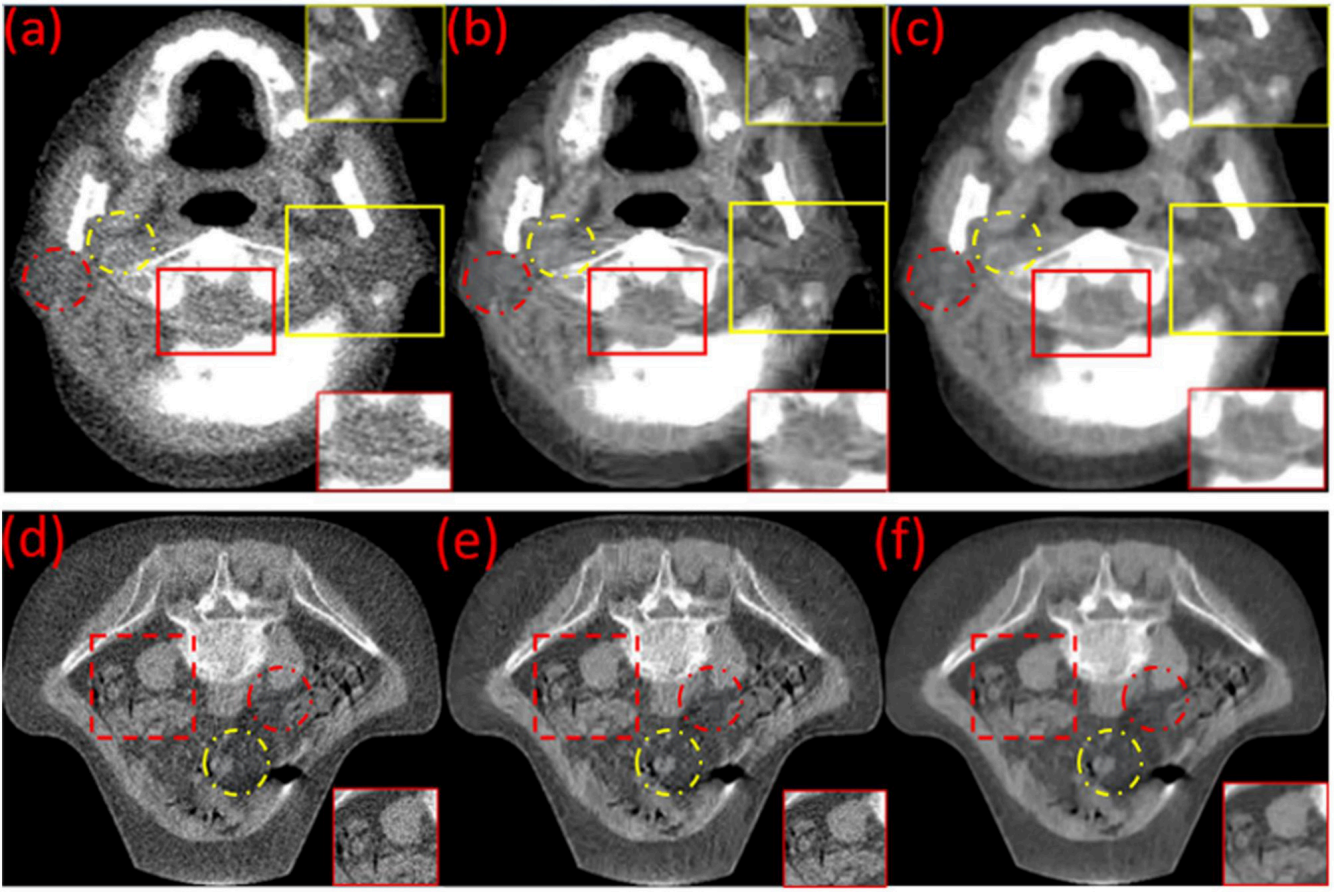
Original **(a,d)** and the post-processed MVCT of (the) clinical head & neck and pelvic images by BM3D **(b,e)** and the proposed BM3D + DFR method **(c,f)**.

In the head & neck and pelvic MVCT images of Figure 4, the red dotted circles represent the target regions, and the yellow dotted circles represent the background regions for the calculation of the CNR. The noise characteristics suppress the performance of some small feature characteristics in Figures 4a,d. Boundaries for the muscles and intestinal canals are obscured. However, post-processed images (Figures 4b,e) using the BM3D and (Figures 4c,f) BM3D + DFR method, show enhanced contrasts for the soft tissues and improved clarity for bony structures. In the head & neck and pelvic MVCT images of Figure 4, the red box marks the spinal cord and the yellow box marks the parotid gland. The noise characteristics suppress the performance of the spinal cord and the parotid gland in Figure 4a. However, post-processed images (Figures 4b,c) using the BM3D and BM3D + DFR methods, show enhanced contrasts for the soft tissues and an improved clarity for the boundaries of the spinal cord and parotid gland.

### Quantitative Assessment of MVCT Image Enhancement Effects

For both phantom and clinical images, the CNR statistical assessment result of the original and post-processed MVCT serials, using the BM3D and the proposed BM3D + DFR methods on head & neck, chest, and pelvic anatomical sites, are shown in Table 1. It has been interpreted that, the proposed novel BM3D + DFR method significantly improves the image quality on the soft tissue contrast in comparison to both the original and BM3D processed images.

**Table 1:** The contrast-to-noise ratio (CNR) statistical assessment result of the original and post-processed MVCT serials by BM3D and the proposed BM3D + DFR methods on head and neck, chest and pelvic anatomical sites.

	**Original** **(A)**	BM3D^*^ **(B)**	**BM3D + DFR^**§**^** **(C)**	**A vs. B^**¶**^**	**A vs. C**	**B vs. C**
Phantom	1.70	2.90	4.03	-	-	-
Head & neck	1.45 ± 1.51^#^	1.77 ± 1.63	2.09 ± 1.68	*t* = −16.40 *p* < 0.001	*t* = −22.50 *p* < 0.001	*t* = −16.41 *p* < 0.001
Chest	0.92 ± 0.78	1.16 ± 0.75	1.36 ± 0.85	*t* = −4.18 *p* < 0.001	T = −5.69 p < 0.001	*t* = −3.84 *p* < 0.001
Pelvic	1.12 ± 1.22	1.43 ± 1.21	1.76 ± 1.31	*t* = −11.36 *p* < 0.001	*t* = −15.68 *p* < 0.001	*t* = −9.49 *p* < 0.001

* BM3D, Block-Matching 3D-transform;

§ DFR, Discriminative Feature Representation;

¶ t and p value from Paired-Samples T Test;

# *, Average ± Standard Deviation*.

### Evaluation of Density Stability on Post-processed MVCT Serials

**Figure 5:**
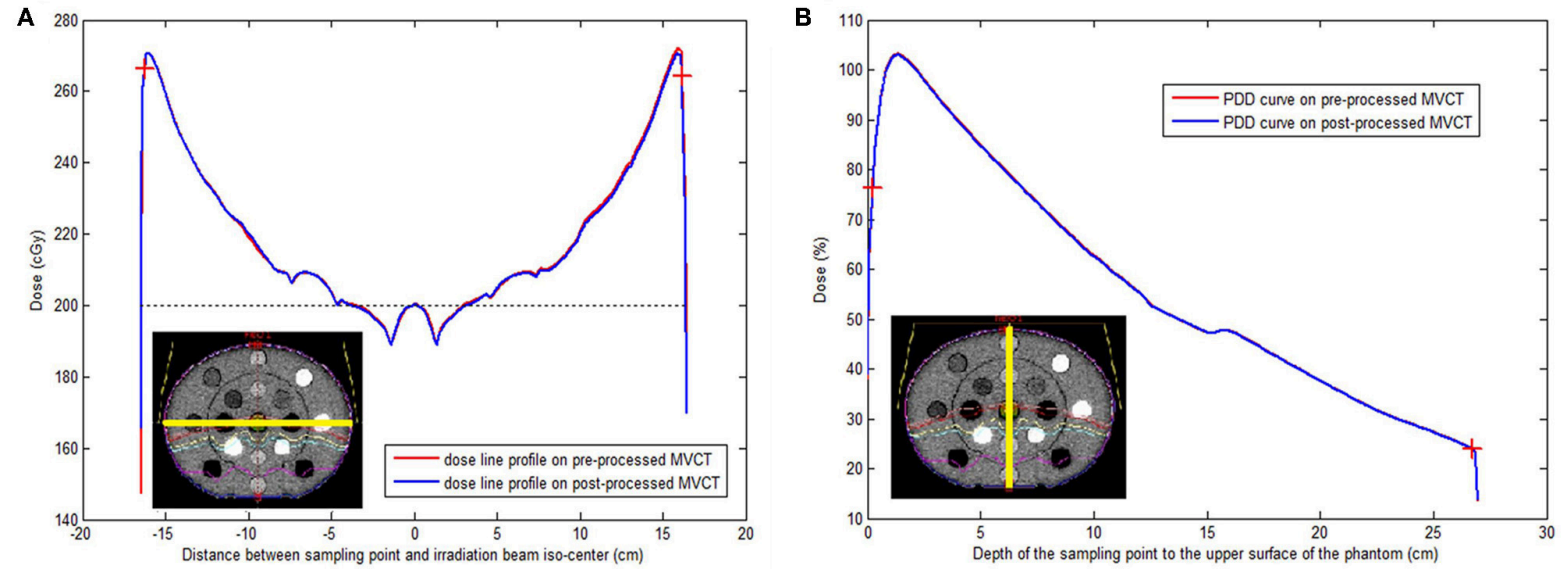
**(A)** Iso-center passed horizontal dose line profiles tested on original (red) and post-processed (blue) MVCT images. **(B)** Iso-center passed vertical percentage depth dose curves on original (red) and post-processed (blue) MVCT images. The yellow lines on the two schematic pictures at both left bottoms showed the position of the dose line profiles **(A)** and percentage depth dose curves **(B)** on the CT-to-electron density phantom (lower left corner) with irradiation dose. Red cross: the start and ending test point for RMS calculation.

Both simulated irradiation plans on the original and BM3D + DFR processed MVCT delivered 308 MU, which means that the dose normalization points (iso-center) were crossed by 200 cGy dose curves. Dose sampling points on the horizontal profile curve (along the yellow line in Figure 5A) were compared between the original and post-processed MVCT, where the sampling points started from the left to the right edge on the transversal slice, crossing the iso-center with a 0.03 cm sample distance and 1,316 sample points. Dose sampling points on the vertical PDD curves (along the yellow line in Figure 5B) were also compared, where the sampling points started from the top to the bottom edge on the transversal slice crossing the iso-center with 0.03 cm sample distance and 1,079 sample points. The calculation starting and ending points were marked on the plot Figures with red crosses on Figures 5A,B. Figure 5 shows that, for both dose line profiles and PDD curves, the dosimetry sampling points calculated on both the original and post-processed MVCT images overlap, which indicates that the post-processed MVCT keeps the density information stable. The RMS values of the dose distribution difference on the horizontal dose profile curves and vertical PDD curves were 0.09% and 0.06%, respectively. This validates the capability of the post-processed MVCT to be used for ART dose calculation.

## Discussion

### Tomo MVCT Enhancement Asks for the Post-reconstructed Image Processing Method

To our knowledge, the existing methods for improving CT image quality may be roughly classified into three categories: data projection correction method, iterative reconstruction method and post-reconstructed method. The first two categories rely on projection data available from the CT equipment. Regarding the MVCT from TomoTherapy, CTrueTM IR (Accuray, Madison WI, USA) has been applied in the new TomoTherapy platform of Radixact®, which aims to reduce MVCT image noise and provide better soft-tissue contrast, while maintaining the same radiation dose. However, considering the IR (iterative reconstruction) method requires projections generated during the MVCT imaging process, its application might be limited for the unavailability of the raw projection data. An iterative reconstruction algorithm is computationally intensive to solve optimization problems, which may be time consuming and inappropriate for on-line image (like Tomo MVCT) enhancement. Post-reconstructed methods, like the traditional DFR and the proposed BM3D + DFR, can be directly used on CT images after 3D reconstruction without the necessity for projection data, which provides more flexibility for the image enhancement.

Except for the BM3D + DFR method proposed in our study and literature mentioned above (6–8), some other post-reconstructed solutions may also provide options for MVCT enhancement. A deep learning method based on 2D and 3D residual convolutional networks was proposed for LDCT enhancement, and this method was proven to maintain high image quality and to reduce both noise and artifacts effectively, while preserving tissue details (18). A similar conclusion was also drawn by Kang et al. who also proposed a LDCT image enhancement solution using a deep learning technique (19). It is considered that deep learning can reveal connotative relations between original obscure images (such as images acquired from MVCT) and the clear real images (such as images acquired from diagnostic KVCT) without manually extracted subjective features in massive images. With the increased image data base and powerful artificial neural network, this category of methods is promising, especially in the era of artificial intelligence.

The construction of a discriminative feature dictionary is a critical step for the DFR method, which will directly affect the image enhancement effect. Different to the application of DFR on LDCT (9), which is acquired on the same CT equipment with different protocols to HDCT, MVCT and planning KVCT were acquired on different equipment with different X-rays at different times. Considering the patient's shape change during planning KVCT and MVCT scanning, it is impossible to construct a discriminative feature dictionary where the pixel-to-pixel absolutely strictly correspond. Therefore, the effect of BM3D + DFR on MVCT is not the same as the effect of DFR on LDCT. However, if considerable MVCT-KVCT corresponding images, on rigid phantoms or MVCT-KVCT deformable registration techniques, can be enrolled in discriminative feature dictionary construction, the BM3D + DFR effect will be improved directly and significantly.

### Enhanced MVCT May Play More Important Role Than IGRT

MVCT images can not only be used for position correction in IGRT, but also for ART dose calculation. Considering MVCT has a reliable CT number to electron density calibration curve, MVCT has been proven accurate for the use of calculating an adaptive daily dose distribution, which is an assurance of ART (20). MVCT consisted of advantages in the lower absorption dose and a larger imaging capacity (theoretically 40 cm reconstruction field of view × 160 cm longitudinal scanning length) than the C-arm based CBCT technology; thus making daily MVCT imaging safe in assessing patient treatment locations and making on-line adaptive re-planning possible using the same MVCT data sets (2). Therefore, the proposed BM3D + DFR method should keep the CT number of MVCT stable without decreasing the dosimetry calculation accuracy. This study checked this issue from both macro (with DVH) and micro (with 0.03 cm sample distance on dose distribution map) perspectives, and finally confirmed the stability of the proposed BM3D + DFR method on dose calculation.

MVCT can also be used in prognosis prediction and radiomic researches. Assisted by MVCT, the study from Bral (21) prospectively assess the feasibility, toxicity, and local control of a class solution protocol of moderately hypofractionated tomotherapy in Stage III, inoperable, locally advanced non–small-cell lung cancer patients. In that study, MVCT played critical role in not only adaptive tumor assessment for review and analysis of primary tumor volume regression, but also toxicity prediction on the suspicion of radiopneumonitis. Consider if MVCT can present more clear view especially on the soft tissue contrast, daily MVCT on different anatomical sites, such as head & neck, abdomen, and pelvic sites with majority of soft-tissues, post-processed MVCT may bring more benefits on prognosis and toxicity predictions.

Our previous study (22, 23) proposed that, with texture features extracted by Radiomic techniques, online cone beam CT images may be used to predict radiotherapy prognosis, whose prediction accuracy was higher than the existing clinical standards like RECIST. The effectiveness of radiomics techniques has also been demonstrated on predicting radiotherapy efficacy, complications, and prognosis (24–26). Considering that post-processed MVCT images offer a more accurate tumor and soft tissue delineation, with the MVCT-KVCT registration technique, the Radiomics prediction on CT may be more specific with certain tumors or organs. Furthermore, considering that our recent research has proven that the MVCT has higher texture feature reproducibility than CBCT (23), even the MVCT may be employed to extract radiomics texture features at prognosis and toxicity prediction in the future.

### Limitation of the BM3D + DFR Method for MVCT Enhancement

The defect of the proposed BM3D + DFR method is that, atoms with the same features will reduce the discriminative features between the KVCT and the difference image, which is produced by the KVCT minus the MVCT images. This reduction in features will cause noise residue in the MVCT images. Some normal tissue structure information may be replaced in the post-processed MVCT, by noise atoms. To solve this problem, the learning morphological diversity (27) and Fisher Discrimination Dictionary Learning (FDDL) (28) methods should be used to increase feature diversity and to maximize feature differences (between two discriminative sub-dictionaries). Further studies are expected with these methods.

## Conclusion

MVCT images of one phantom and patients were post-processed using BM3D and the novel proposed BM3D + DFR combination method. The proposed BM3D + DFR method can feasibly improve the soft tissue contrast of the MVCT image on the head & neck, chest, and pelvic anatomical sites. Furthermore, compared with the original MVCT, it was accompanied by a dose calculation without compromising the resolution. After integration in clinical workflow, the post-processed MVCT may be better applied in image-guided and adaptive helical TomoTherapy.

## Ethics Statement

This work was approved by the ethics committee of the Shandong Cancer Hospital, Affiliated to Shandong University. The need for informed consent was waived by the Medical Ethics Committee because the study was an observational, retrospective study using an image database from which the patients' identifying information had been removed.

## Author Contributions

JZ designed the study and contributed to its conception. JZ and YL were major contributors in the writing of the manuscript. CY performed the previous experiments using the DFR method. HY and YC are engineers who acquired and organized the original MVCT images. YY and BL checked the experimental data and provided advice. JD revised the manuscript for important intellectual content. All authors read and approved the final manuscript.

### Conflict of Interest Statement

The authors declare that the research was conducted in the absence of any commercial or financial relationships that could be construed as a potential conflict of interest.
